# Channel Modeling and Characteristics Analysis under Different 3D Dynamic Trajectories for UAV-Assisted Emergency Communications

**DOI:** 10.3390/s23125372

**Published:** 2023-06-06

**Authors:** Jingfan Zhang, Yu Liu, Jie Huang, Hengtai Chang, Zhaolei Zhang, Jingquan Li

**Affiliations:** 1 School of Microelectronics, Shandong University, Jinan 250101, China; 202132424@mail.sdu.edu.cn (J.Z.); zhaoleizhang@mail.sdu.edu.cn (Z.Z.); 202232357@mail.sdu.edu.cn (J.L.); 2 The National Mobile Communications Research Laboratory, School of Information Science and Engineering, Southeast University, Nanjing 210096, China; j_huang@seu.edu.cn (J.H.); changhengtai@pmlabs.com.cn (H.C.); 3 The Purple Mountain Laboratories, Nanjing 211111, China

**Keywords:** multi-trajectory, multi-mobility, UAV-assisted emergency communications, Markov chain

## Abstract

This study involved channel modeling and characteristics analysis of unmanned aerial vehicles (UAVs) according to different operating trajectories. Based on the idea of standardized channel modeling, air-to-ground (AG) channel modeling of a UAV was carried out, taking into consideration that both the receiver (Rx) and the transmitter (Tx) ran along different types of trajectories. In addition, based on Markov chains and a smooth-turn (ST) mobility model, the influences of different operation trajectories on typical channel characteristics—including time-variant power delay profile (PDP), stationary interval, temporal autocorrelation function (ACF), root mean square (RMS) delay spread (DS), and spatial cross-correlation function (CCF)—were studied. The multi-mobility multi-trajectory UAV channel model matched well with actual operation scenarios, and the characteristics of the UAV AG channel could be analyzed more accurately, thus providing a reference for future system design and sensor network deployment of sixth-generation (6G) UAV-assisted emergency communications.

## 1. Introduction

With the further promotion of commercialization of fifth-generation (5G) communications, research on sixth-generation (6G) communications has also begun. With the further upgrade of the communication network, 6G communications will cover more application fields, and will meet the needs of more application scenarios, which will bring higher transmission rates, lower delay, larger network capacity, and lower network energy consumption [1,2,3,4,5]. To build an air–space–ground–sea integrated communication network, and to meet the communication needs of more complicated scenarios, unmanned-aerial-vehicle-(UAV) communications are still essential. UAVs have the advantages of convenient deployment, flexible mobility, and low cost, which make them an important tool for assisting ground communications [6], especially emergency communications. In order to better design the air-to-ground (AG) communication system and optimization wireless sensor network of UAVs, the AG channel model needs to be more practical, to mimic real channel characteristics.

Based on the standardized channel model, modeling of typical communication scenarios has been carried out, such as high-speed train (HST) scenarios, maritime scenarios, underground scenarios, etc. With the further optimization of the HST channel model, research on space–time–frequency (STF) domain channel characteristics has become increasingly abundant. A three-dimensional (3D) millimeter-wave (mmWave) channel model, taking into consideration STF massive multiple-input, multiple-output (MIMO), was proposed in article [7], and some typical channel characteristics—such as temporal autocorrelation function (ACF), delay power spectral density (PSD), spatial cross-correlation function (CCF), angular PSD, and frequency correlation function (FCF)—were studied. Based on the spatial and temporal correlation of the MIMO channels of an HST, a spatial–temporal channel prediction model, introducing a convolutional neural network (CNN) and convolutional long short-term memory (CLSTM), was presented [8]. For the purpose of meeting the demands of future terahertz-(THz)-band communications in smart HST scenarios, the related channel characteristics for train-to-infrastructure (T2I) were analyzed, based on ray-tracing (RT) simulators and actual measurement data [9]. The performance of THz communications under different antenna patterns and weather conditions was investigated, based on channel capacity, in article [10]. Waveguide effects at the sea surface became a research focus topic when the research area shifted to maritime channel modeling. A non-stationary channel model for 3D UAV-to-ship was proposed in article [11], considering the line of sight (LoS) component, the single bounce (SB) component due to sea surface fluctuations, and the multiple bounce (MB) component due to sea surface waveguide effects. The rapid advancement of UAVs has placed them in an advantageous position in auxiliary communications, due to their high mobility and ease of deployment [12]. A UAV-assisted search and location strategy was proposed, to locate victims in disaster non-LoS (NLoS) situations [13]. Article [14] proposed a UAV-to-vehicle (U2V) mmWave communication channel model, to research the relevant channel characteristics. For vehicle communications in harsh environments, a UAV as a mobile base station (BS) can achieve better wireless services [15].

At present, there are two main methods for the research of the UAV AG channel: one is based on an empirical channel model according to measurement data; the other is based on a deterministic or geometric channel model according to simulation data [16]. To better meet the needs of future 6G application communications, the measurement scenarios of UAV AG communications are mostly focused on cities, suburban areas, rural areas, and open areas. Some key statistical properties, such as large-scale fading or small-scale fading, have been analyzed in these areas [17]. Measurements of an AG communication channel, at 2.585 GHz and 3.5 GHz, based on different trajectories of UAVs, were conducted in hilly scenarios [18], where shadow fading (SF), path loss (PL), root mean square (RMS) delay spread (DS), and other characteristics of the UAV AG channel were analyzed and compared to the measured data. Measurements of the AG channel were conducted at C-band and L-band, in multiple scenarios, in [19,20,21], including near-urban, hilly, over-water, suburban, and mountainous environments. Through the measured data, the most direct AG statistical properties of the different terrains were obtained. Moreover, the effect of airframe shadowing on the AG channel was further investigated, and the method of deploying multiple antennas to help mitigate this effect was validated [22]. In [23], AG channel measurements were conducted at multiple frequencies, and then a novel shadow fading model was proposed. The relevant small-scale fading characteristics were also verified by the measurement data. Channel measurements in some specific scenarios are also ongoing. Measurements were conducted in a linear subway tunnel at 1.8 and 5.8 GHz, resulting in some large-scale channel characteristics [24]. Measurements for vehicle-to-vehicle (V2V), at 5.9 GHz, based on street intersection scenarios, were carried out, and some measurement data were provided [25]. Although measurement data can provide first-hand channel characteristics, some measurement environments are complex, and the measurement cost is high; therefore, using a deterministic or stochastic channel model of simulation data can more easily simulate the UAV AG communication channel. A general 5G channel model for small-scale fading characteristics, based on the WINNER II and Saleh–Valenzuela (SV) channel models, has been proposed. Moreover, the cluster evolution on the time axis has been investigated [26]. The study of small-scale fading is extremely important for UAV AG communication channels. A GBSM with 3D dynamic trajectories at the transmitter (Tx) and the fixed receiver (Rx) was studied in article [27]. Some important channel characteristics, such as power delay profile (PDP), stationary interval, space–time correlation function (STCF), Doppler PSD, RMS DS, and RMS Doppler spread, were analyzed. Taking into consideration level crossing rate (LCR), STCF, Doppler PSD, and average fade duration (AFD), article [28] proposed a regular-shaped, geometry-based statistical model (RS-GBSM) for a UAV MIMO channel. The statistical properties of the channel were derived, based on the effects of parameters such as flight altitude and direction of movement. A model with mixed bouncing was proposed, to capture AG channel characteristics [29]. A general channel model framework, which was suitable for multi-frequencies and multi-scenarios, was proposed [30]. Taking into consideration the changing moving direction and speed of the MS, a channel model of 3D wideband MIMO was proposed [31], and the statistical properties were verified by simulation data and theoretical data. In order to capture the effects of objects and human movement, a 3D channel model for a moving point scatterer in indoor scenarios was proposed in paper [32]. To obtain the real-time azimuth angle of departure (AAoD), the elevation angle of departure (EAoD), the azimuth angle of arrival (AAoA), and the elevation angle of arrival (EAoA), a novel angle estimation algorithm was presented in a 3D MIMO channel model for AG communications [33].

As far as the authors know, current research on the UAV AG channel model mainly focuses on the channel characteristics of fixed BSs, while research on AG multi-mobility multi-trajectory channel characteristics is still lacking. In some typical UAV-assisted communication scenarios—especially emergency communication scenarios—there is a higher randomness in the trajectory of the Tx and the Rx. Not only can the trajectory of UAV-assisted emergency communications affect the quality of the communication link, but also, too much ineffective movement can increase energy consumption; therefore, the effect of different trajectories of the Rx and the Tx on the channel characteristics needed to be studied. Based on the above status, we propose a multi-mobility multi-trajectory channel model, which can make the AG channel model more consistent with actual scenarios. The main contributions and novelties of this article are as follows:

1. For this study, our proposed model took two approaches to realizing multi-trajectories. A Markov chain was introduced in the aerial part, by changing the azimuth and elevation angle of the UAV flight, to obtain a random trajectory. It could simulate the dive, climb, and level flight of the UAV in arbitrary 3D space. As most vehicles on the ground-Rx end do not make sharp turns, we used a smooth-turn (ST) mobility model to simulate the movement of the Rx end in two dimensions. Moreover, the trajectories of moving clusters were also considered, to mimic the movement of vehicles around the Rx.
2. Based on the proposed channel model, typical AG channel characteristics of UAV communications during different trajectories of the Tx and the Rx were studied, including PDP, temporal ACF, spatial CCF, stationary interval, and RMS DS. By analyzing and studying the effect of multi-mobility multi-trajectories on statistical properties, the non-stationary characteristics of the UAV AG channel were analyzed and compared.
3. Channel measurement of relevant scenarios was carried out. Some statistical properties of the proposed channel model were verified by actual measurement data, which demonstrated the accuracy of the proposed model.

The remainder of this paper is structured as follows. In Section 2, a UAV-assisted communication network, with multi-mobility multi-trajectory cases, is introduced. UAV AG communication channel modeling and characterization are studied in Section 3. Numerical results and analysis are provided in Section 4. Conclusions are drawn in Section 5.

## 2. UAV-Assisted Communication Network with Multi-Mobility Multi-Trajectory Cases

**Figure 1:**
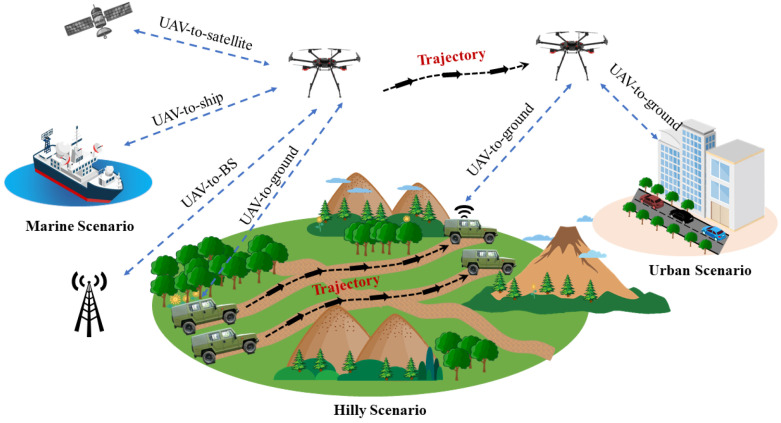
UAV-assisted emergency communication scenarios.

UAV communications, as an important component of the space–air–ground–sea integrated communication network, can play an important role in various emergency communication scenarios. As shown in Figure 1, the flexible deployment of a UAV in the air can make it a mobile BS, and can provide wireless communication for ground BSs, end users, ships at sea, and satellites. UAV-assisted communications can provide an effective guarantee for maritime communications and ground wireless communications, especially in military and urban transportation emergency communications; the wireless channel between the ground user and the BS is complex and full of interference, which can lead to poor communication quality. In military operations, the ground BS is easily destroyed, and the signal cover range of the BS is limited. UAVs, as aerial BSs for auxiliary communications, can provide effective communication support for ground users, and can also move flexibly, to eliminate communication blind zones caused by lack of ground BSs, and by complex mountain environments. With regard to urban transportation emergency communications, because fixed ground BSs cannot meet sufficient and sudden communication needs, the simple deployment and abundant number of UAVs can realize the emergency communication needs of a large number of users on the ground.

## 3. UAV AG Communication Channel Modeling and Characterization

### 3.1. A UAV AG Multi-Mobility Multi-Trajectories Channel Model

**Figure 2:**
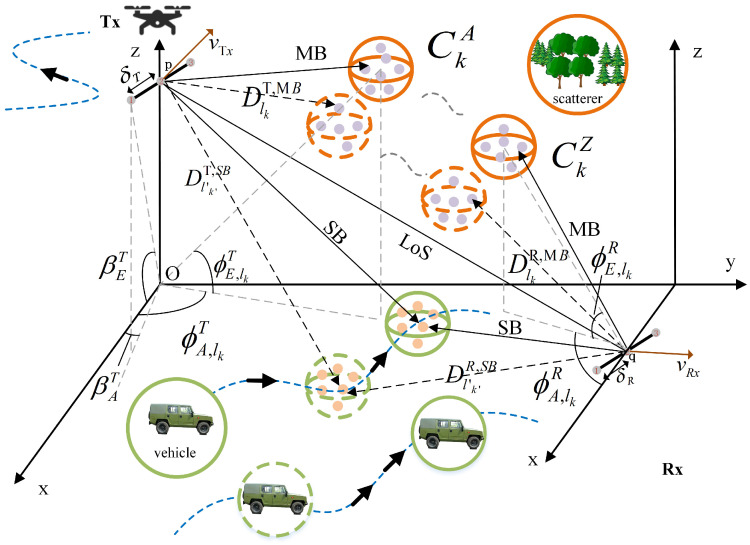
A 3D non-stationary GBSM with multi-trajectories.

The propagation channel between a UAV and a ground BS is affected by large-scale fading and small-scale fading: for this paper, we mainly studied the influence of the latter. Small-scale fading is mainly caused by the multipath components (MPCs) of the channel between the UAV and the vehicle, which can be abstracted as lots of static and moving clusters. The transmission signal between the UAV and the vehicle consists of LoS components and NLoS components, and the NLoS components contain SB components and MB components. Figure 2 gives the model framework of a 3D non-stationary GBSM abstracted by an AG channel with multi-mobility multi-trajectories. The representation of angles, distances, and other parameters is basically the same for the SB case and the MB case; therefore, in order to simplify the content, our article basically shows the parameters of the MB case. The subscripts l,k and $l^{\prime },k^{\prime }$ were used to represent the MB case and the SB case, respectively. In particular, the distances of the ray between the Tx/Rx and the clusters were distinguished, using the superscript MB and SB.

The UAV was set as the Tx, and the vehicle was set as the Rx. For the MB case, the AAoD and EAoD of the *l*th ray in cluster $C_{k}^{A}\left(t\right)$ transmitted from the Tx were denoted by $\phi_{A,l_{k}}^{T}\left(t\right)$ and $\phi_{E,l_{k}}^{T}\left(t\right)$, respectively. $\phi_{A,l_{k}}^{R}\left(t\right)$ and $\phi_{E,l_{k}}^{R}\left(t\right)$ were defined as the AAoA and EAoA of the *l*th ray in cluster $C_{k}^{Z}\left(t\right)$ transmitted from Rx, respectively. The relevant angles of the antenna array are also provided, i.e., $\beta_{E}^{T}$ was the elevation angle, and $\beta_{A}^{T}$ was the azimuth angle. The angle of the ground Rx end was the same as the above, and is not shown in the figure, due to the low height of the Rx end.

The channel coefficients for each cluster and each Tx and Rx element pair were characterized by an $N_{T}$ × $N_{R}$ matrix $H_{small}={\left[h_{pq}\left(t,\tau \right)\right]}_{N_{T}\times N_{R}}$. The channel impulse response (CIR) between the *p*th Tx and the *q*th Rx was represented by LoS and NLoS components, expressed as

(1) $$h_{pq}\left(t,\tau \right)=\sqrt{\frac{K_{R}}{K_{R}+1}}h_{pq}^{LoS}\left(t,\tau \right)+\sqrt{\frac{1}{K_{R}+1}}\left(h_{pq,SB}^{NLoS}\left(t,\tau \right)+h_{pq,MB}^{NLoS}\left(t,\tau \right)\right)$$

where $K_{R}$ was the K-factor, and τ was the time delay. The NLoS components $h_{pq}^{NLoS}\left(t,\tau \right)$ were expressed as

(2) $$\begin{array}{cc} h_{pq,SB}^{NLoS}\left(t,\tau \right)= & \sum_{k^{\prime }=1}^{K_{pq}^{\prime }\left(t\right)}\sum_{l^{\prime }=1}^{L_{k^{\prime }}^{\prime }}{\left[\begin{array}{c} F_{q,V}\left(\phi_{E,l_{k^{\prime }}^{\prime }}^{R}\left(t\right),\phi_{A,l_{k^{\prime }}^{\prime }}^{R}\left(t\right)\right)\\ F_{q,H}\left(\phi_{E,l_{k^{\prime }}^{\prime }}^{R}\left(t\right),\phi_{A,l_{k^{\prime }}^{\prime }}^{R}\left(t\right)\right) \end{array}\right]}^{T}\left[\begin{array}{cc} e^{j\theta_{l^{\prime }k^{\prime }}^{VV}} & \sqrt{\kappa_{l_{k^{\prime }}^{\prime }}^{-1}}e^{j\theta_{l^{\prime }k^{\prime }}^{VH}}\\ \sqrt{\kappa_{l_{k^{\prime }}^{\prime }}^{-1}}e^{j\theta_{l^{\prime }k^{\prime }}^{HV}} & e^{j\theta_{l^{\prime }k^{\prime }}^{HH}} \end{array}\right]\\ \times & \left[\begin{array}{c} F_{p,V}\left(\phi_{E,l_{k^{\prime }}^{\prime }}^{T}\left(t\right),\phi_{A,l_{k^{\prime }}^{\prime }}^{T}\left(t\right)\right)\\ F_{p,H}\left(\phi_{E,l_{k^{\prime }}^{\prime }}^{T}\left(t\right),\phi_{A,l_{k^{\prime }}^{\prime }}^{T}\left(t\right)\right) \end{array}\right]\sqrt{P_{pq,l_{k^{\prime }}^{\prime }}\left(t\right)}e^{j2\pi f_{c}\left(\left(D_{p,l_{k^{\prime }}^{\prime }}^{T,SB}\left(t\right)+D_{q,l_{k^{\prime }}^{\prime }}^{R,SB}\left(t\right)\right)/c+\tilde{\tau }\right)}\\ \cdot & \delta (\tau -\left(\left(D_{p,l_{k^{\prime }}^{\prime }}^{T,SB}\left(t\right)+D_{q,l_{k^{\prime }}^{\prime }}^{R,SB}\left(t\right)\right)/c+\tilde{\tau }\right)) \end{array}$$

(3) $$\begin{array}{cc} h_{pq,MB}^{NLoS}\left(t,\tau \right)= & \sum_{k=1}^{K_{pq}\left(t\right)}\sum_{l=1}^{L_{k}}{\left[\begin{array}{c} F_{q,V}\left(\phi_{E,l_{k}}^{R}\left(t\right),\phi_{A,l_{k}}^{R}\left(t\right)\right)\\ F_{q,H}\left(\phi_{E,l_{k}}^{R}\left(t\right),\phi_{A,l_{k}}^{R}\left(t\right)\right) \end{array}\right]}^{T}\left[\begin{array}{cc} e^{j\theta_{lk}^{VV}} & \sqrt{\kappa_{l_{k}}^{-1}}e^{j\theta_{lk}^{VH}}\\ \sqrt{\kappa_{l_{k}}^{-1}}e^{j\theta_{lk}^{HV}} & e^{j\theta_{lk}^{HH}} \end{array}\right]\\ \times & \left[\begin{array}{c} F_{p,V}\left(\phi_{E,l_{k}}^{T}\left(t\right),\phi_{A,l_{k}}^{T}\left(t\right)\right)\\ F_{p,H}\left(\phi_{E,l_{k}}^{T}\left(t\right),\phi_{A,l_{k}}^{T}\left(t\right)\right) \end{array}\right]\sqrt{P_{pq,l_{k}}\left(t\right)}e^{j2\pi f_{c}\left(\left(D_{p,l_{k}}^{T,MB}\left(t\right)+D_{q,l_{k}}^{R,MB}\left(t\right)\right)/c+\tilde{\tau }\right)}\\ \cdot & \delta (\tau -\left(\left(D_{p,l_{k}}^{T,MB}\left(t\right)+D_{q,l_{k}}^{R,MB}\left(t\right)\right)/c+\tilde{\tau }\right)) \end{array}$$

where ${\left\{\cdot \right\}}^{T}$ denoted the transpose operation, $f_{c}$ was the carrier frequency, and $F_{p/q,V}$ and $F_{p/q,H}$ denoted the antenna patterns of vertical and horizontal polarizations at the Tx or the Rx, respectively. Moreover, $\kappa_{l_{k}}$ represented the cross-polarization power ratios (XPR) of the *l*th ray in cluster $C_{k}^{A/Z}$; and $\theta_{l_{k}}^{Tr}$ (where Tr respected VV,VH,HV,HH) represented initial phases subject to uniform distribution in $\left(0\right.,\left.2\pi \right]$; while $D_{p,l_{k}}^{T,MB}\left(t\right)$ and $D_{q,l_{k}}^{R,MB}\left(t\right)$ were the distance of *l*th ray from the *p*th Tx antenna element to the scattering cluster $C_{k}^{A}\left(t\right)$, and the distance of the *l*th ray from the scattering cluster $C_{k}^{Z}\left(t\right)$ to the *q*th Rx antenna element, respectively; $P_{pq,l_{k}}\left(t\right)$ were denoted as the powers of the *l*th ray in the *k*th cluster between the Tx and the Rx at time *t*; *c* was the speed of light; and $\tilde{\tau }$ was the time delay of the virtual link.

The LoS component was expressed as

(4) $$\begin{array}{cc} h_{pq}^{LoS}\left(t,\tau \right)= & {\left[\begin{array}{c} F_{q,V}\left(\phi_{E,LoS}^{R}\left(t\right),\phi_{A,LoS}^{R}\left(t\right)\right)\\ F_{q,H}\left(\phi_{E,LoS}^{R}\left(t\right),\phi_{A,LoS}^{R}\left(t\right)\right) \end{array}\right]}^{T}\left[\begin{array}{cc} e^{j\theta_{LoS}^{VV}} & 0\\ 0 & e^{j\theta_{LoS}^{HH}} \end{array}\right]\left[\begin{array}{c} F_{p,V}\left(\phi_{E,LoS}^{T}\left(t\right),\phi_{A,LoS}^{T}\left(t\right)\right)\\ F_{p,H}\left(\phi_{E,LoS}^{T}\left(t\right),\phi_{A,LoS}^{T}\left(t\right)\right) \end{array}\right]\\ \times & e^{j2\pi f_{c}D_{pq}^{LoS}\left(t\right)/c}\cdot \delta \left(\tau -D_{pq}^{LoS}\left(t\right)/c\right) \end{array}$$

where $D_{pq}^{LoS}\left(t\right)$ was the distance between the *p*th Tx antenna element and the *q*th Rx antenna element.

The calculations of $D_{p,l_{k}}^{T,MB}\left(t\right)$, $D_{q,l_{k}}^{R,MB}\left(t\right)$, and $D_{pq}^{LoS}\left(t\right)$ were expressed as

(5) $$D_{p,l_{k}}^{T,MB}\left(t\right)=\left|\right|C_{l_{k}}^{A}\left(t\right)-A_{p}^{T}\left(t\right)\left|\right|$$

(6) $$D_{q,l_{k}}^{R,MB}\left(t\right)=\left|\right|A_{q}^{R}\left(t\right)-C_{l_{k}}^{Z}\left(t\right)\left|\right|$$

(7) $$D_{pq}^{LoS}\left(t\right)=\left|\right|A_{q}^{R}\left(t\right)-A_{p}^{T}\left(t\right)\left|\right|$$

where $C_{l_{k}}^{A}\left(t\right)$ and $C_{l_{k}}^{Z}\left(t\right)$ were coordinates of the scattering points of the *l*th ray in the cluster $C_{k}^{A}\left(t\right)$ and $C_{k}^{Z}\left(t\right)$, respectively. Furthermore, $A_{q}^{R}\left(t\right)$ and $A_{p}^{T}\left(t\right)$ were the coordinates of the *q*th Rx antenna element, and the coordinates of the *p*th Tx antenna element, respectively.

The delay of the rays varied with time *t*. The cluster power satisfied an exponential distribution with a single slope, and the total power was set to 1 by a normalization operation [27]. $P_{pq,l_{k}}\left(t\right)$ changed with the moving trajectory. Here, we set different trajectories at the Tx and the Rx, respectively.

The parameters involved in the channel modeling are listed in Table 1.

#### 3.1.1. Generation of Tx Trajectories

To enhance the randomness of the model, Markov chains were introduced, by setting random azimuths as well as elevation angles to simulate UAV arbitrary trajectories. Markov chains are stochastic processes in the state space, which undergo transitions from one state to another [34]. The process requires “memorylessness”, i.e., the probability distribution of the next state can only be determined by the current state, and the events preceding it in the time series are irrelevant. The process contains three elements: state space; transfer matrix; and initial state. A sequence of random variables $X=\left\{X_{0},X_{1},\cdots ,X_{i},\cdots \right\}$, $X_{n}$ depends only on $X_{i-1}$, i.e.,

(8) $$P\left(X_{i}|X_{0},X_{1},\cdots ,X_{i-1}\right)=P\left(X_{i}|X_{i-1}\right),i=1,2,\cdots$$

where $P\left(X_{i}|X_{i-1}\right)$ is the transition probability distribution. The flight angle of the UAV was determined by the azimuth angle α and the elevation angle γ, and we used a Markov chain to randomly generate the azimuth and elevation angles. $A_{p}^{T}\left(t\right)$ was expressed as

(9) $$A_{p}^{T}\left(t\right)=A_{ini,p}^{T}+V_{P}t,$$

where $A_{ini,p}^{T}\left(t\right)$ was the initial coordinate of the *p*th Tx antenna, $A_{p}^{T}\left(t\right)$ was the coordinate of the *p*th Tx antenna, after introducing a Markov chain, α was in $\left[\alpha_{\mathrm{ini}},\alpha_{\mathrm{end}}\right]$, and γ was in $\left[\gamma_{\mathrm{ini}},\gamma_{\mathrm{end}}\right]$. The moving range of the UAV was controlled by the azimuth angles $\alpha_{\mathrm{ini}}$, $\alpha_{\mathrm{end}}$ and by the elevation angles $\gamma_{\mathrm{ini}}$, $\gamma_{\mathrm{end}}$. By setting the values of $\alpha_{\mathrm{ini}}$, $\alpha_{\mathrm{end}}$, $\gamma_{\mathrm{ini}}$, $\gamma_{\mathrm{end}}$, the moving range of the UAV could be adjusted. The speed vector of the Tx $V_{P}$ was expressed as

(10) $$V_{P}=\left[\begin{array}{c} v_{P,x}\\ v_{P,y}\\ v_{P,z} \end{array}\right]=\left[\begin{array}{c} V_{p,x}cos\alpha sin\gamma \\ V_{p,y}sin\alpha sin\gamma \\ V_{p,z}cos\gamma \end{array}\right].$$

**Figure 3:**
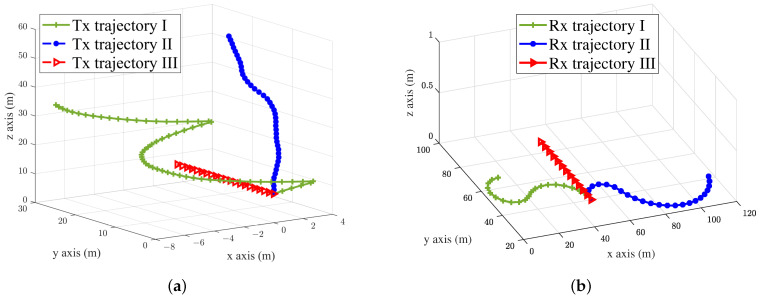
Simulation of the Tx and Rx trajectories: (**a**) Tx for different trajectories based on Markov chain; (**b**) Rx for different trajectories based on ST mobility model.

The initial state probabilities were all 1/$\left(\alpha_{end}-\alpha_{ini}\right)$, and the initial transition matrix was subject to Poisson distribution. In the case of equal time, a Markov chain was used to obtain the state chain in turn. After multiple screening of the simulation, the following two random trajectories were obtained, as shown in Figure 3.

#### 3.1.2. Generation of Rx Trajectories

In the daily ground driving process, vehicles have fewer sharp turns and sharp driving routes; therefore, in order to better simulate the trajectory of the Rx movement, a ST mobility model was introduced [35]. The basic principle of the model is that the vehicle moves in a circle along a point in a straight line perpendicular to the forward direction, until the next steering center is selected. The process of the basic ST mobility model during the time interval $T_{i}\leq t<T_{i+1}$ was as follows:

(11) $$a_{t,i}\left(t\right)=0$$

(12) $$a_{n,i}\left(t\right)=\frac{V^{2}}{r\left(T_{i}\right)}$$

(13) $$\dot{\Phi }\left(t\right)=-\omega \left(t\right)=-\frac{V}{r\left(T_{i}\right)}$$

(14) $$v_{Q,x}\left(t\right)=V\cos\left(\Phi \left(t\right)\right)$$

(15) $$v_{Q,y}\left(t\right)=V\sin\left(\Phi \left(t\right)\right),$$

where the “.” was the first-order derivative with respect to time t. The horizontal tangential and centripetal acceleration were denoted as $a_{t},i\left(t\right)$ and $a_{n},i\left(t\right)$, respectively. The velocity along the x and y axes during the time interval $T_{i}\leq t<T_{i+1}$ were defined as $v_{Q,x}\left(t\right)$ and $v_{Q,y}\left(t\right)$, respectively. The angle and angular acceleration of circular motion during the time interval $T_{i}\leq t<T_{i+1}$ were denoted as $\Phi \left(t\right)$ and $\omega \left(t\right)$, respectively. The speed vector was V, and the turning radius was $r\left(T_{i}\right)$. The inverse of the turning radius followed a Gaussian distribution with zero mean and variance $\sigma_{s}^{2}$. The turning radii $r\left(T_{i}\right)>0$ represented turning right and $r\left(T_{i}\right)<0$ represented turning left. The time interval $\tau_{i}=T_{i+1}-T_{i}$ was subject to exponential distribution. The coordinates of Rx were expressed as

(16) $$A_{q}^{R}\left(t\right)=A_{ini,q}^{R}+V_{Q}t$$

(17) $$V_{Q}=\left[\begin{array}{c} v_{Q,x}\\ v_{Q,y}\\ v_{Q,z} \end{array}\right]=\left[\begin{array}{c} V_{q,x}\cos\left(\Phi \left(t\right)\right)\\ V_{q,y}\sin\left(\Phi \left(t\right)\right)\\ V_{q,z_{0}} \end{array}\right],$$

where $A_{ini,q}^{R}$ was the initial coordinate of the *q*th Rx antenna. The height of the ground Rx end was constant, and remained $V_{q,z_{0}}$ = 0 m/s.

#### 3.1.3. Generation of Cluster Trajectories

When a ground BS cannot meet the communication requirements of a great deal of vehicles as well as users, a UAV-assisted emergency network is considered. UAVs used as mobile BSs are deployed rapidly, and the AG channel at this time needs to be further investigated: thus, the single cluster model was employed for simulation of an AG channel model for military and urban emergency communication scenarios. It was assumed that the interference of many vehicles near the Rx could be abstracted into multiple clusters; that such clusters would have the same trajectory with the Rx; and that the appearance and disappearance of vehicles in the range were characterized by the birth and death process of clusters; therefore, the coordinates of the cluster were expressed as

(18) $$C_{l_{k^{\prime }}^{\prime }}\left(t\right)=A_{q}^{R}\left(t\right)=A_{ini,q}^{R}+V_{Q}t+\Delta D$$

(19) $$D_{p,l_{k^{\prime }}^{\prime }}^{T,SB}\left(t\right)=\left|\right|C_{l_{k^{\prime }}^{\prime }}\left(t\right)-A_{p}^{T}\left(t\right)\left|\right|$$

(20) $$D_{q,l_{k^{\prime }}^{\prime }}^{R,SB}\left(t\right)=\left|\right|A_{q}^{R}\left(t\right)-C_{l_{k^{\prime }}^{\prime }}\left(t\right)\left|\right|,$$

where $C_{l_{k^{\prime }}^{\prime }}\left(t\right)$ represented the coordinates of a single cluster, $D_{p,l_{k^{\prime }}^{\prime }}^{T,SB}\left(t\right)$ and $D_{q,l_{k^{\prime }}^{\prime }}^{R,SB}\left(t\right)$ were the distance of the $l^{\prime }$th ray between the Tx/Rx and the $k^{\prime }$th cluster, and ΔD was a relative distance.

### 3.2. Typical Statistical Properties of Channel Model

#### 3.2.1. PDP

The time-variant PDP $\Lambda_{pq}\left(t,\tau \right)$, calculated by the power and the delay that the MPCs generated, was denoted as

(21) $$\Lambda_{pq}\left(t,\tau \right)=\sum_{k=1}^{K(t)}\sum_{l=1}^{l_{k}}P_{pq,l_{k}}\left(t\right)\delta \left(\tau -\tau_{l_{k}}\left(t\right)\right).$$

The moving trajectories of the Tx, the Rx, and the cluster changed over time, and the corresponding PDP also changed.

#### 3.2.2. Stationary Interval

The stationary interval is the maximum duration, and the channel statistical properties remain unchanged. The stationary interval was

(22) $$T_{t}\left(t\right)=\max\left\{\Delta t|c\left(t,\Delta t\right)\geq c_{thresh}\right\}$$

where $c_{thresh}$ was a certain threshold, which was set to 0.8. The correlation coefficient c(t,Δt) was shown as

(23) $$c\left(t,\Delta t\right)=\frac{\int \Lambda_{pq}\left(t,\tau \right)\Lambda_{pq}\left(t+\Delta t,\tau \right)d\tau }{\max\{\int \Lambda_{pq}{\left(t,\tau \right)}^{2}d\tau ,\int \Lambda_{pq}{\left(t+\Delta t,\tau \right)}^{2}d\tau \}.}$$

#### 3.2.3. Temporal ACF and Spatial CCF

The temporal ACF, which illustrates the non-stationarity of the channel in the time domain, and the Spatial CCF, which illustrates the non-stationarity of the channel in the space domain, can be deduced from the STCF [27]. The STCF is defined as

(24) $$R_{pq,\tilde{p}\tilde{q}}\left(t;\Delta d,\Delta t\right)=E\left\{h_{pq}\left(t\right)h_{\tilde{p}\tilde{q}}^{*}\left(t-\Delta \left(t\right)\right)\right\}.$$

It contains an LoS part and an NLoS part:

(25) $$R_{pq,\tilde{p}\tilde{q}}\left(t;\Delta d,\Delta t\right)=\frac{K_{R}}{K_{R}+1}R_{pq,\tilde{p}\tilde{q}}^{LoS}\left(t;\Delta d,\Delta t\right)+\frac{1}{K_{R}+1}\sum_{n=1}^{N_{pq}\left(t\right)}R_{pq,\tilde{p}\tilde{q}}^{NLoS}\left(t;\Delta d,\Delta t\right)$$

(26) $$R_{pq,\tilde{p}\tilde{q}}^{LoS}\left(t;\Delta d,\Delta t\right)={\left[P_{pq}^{LoS}\left(t\right)P_{\tilde{p}\tilde{q}}^{LoS}\left(t\right)\right]}^{\frac{1}{2}}\cdot e^{j\frac{2\pi }{\lambda }\left[d_{pq}^{LoS}\left(t\right)-d_{pq}^{LoS}\left(t-\Delta t\right)\right]}$$

(27) $$R_{pq,\tilde{p}\tilde{q}}^{NLoS}\left(t;\Delta d,\Delta t\right)=P_{s}\cdot E\left\{\sum_{l_{k}=1}^{L_{k}}{\left[P_{pq,l_{k}\left(t\right)}\left(t\right)P_{\tilde{p}\tilde{q},l_{k}\left(t\right)}\left(t-\Delta t\right)\right]}^{\frac{1}{2}}\cdot e^{j\frac{2\pi }{\lambda }\left[d_{pq,l_{k}\left(t\right)}\left(t\right)-d_{\tilde{p}\tilde{q},l_{k}\left(t\right)}\left(t-\Delta t\right)\right]}\right\}$$

where Δ*t* is the time interval, Δ*d* is the distance of the antenna, and $P_{s}$ is the joint probability of cluster survival.

#### 3.2.4. RMS DS

The RMS DS is a second-order statistical property, which characterizes the dispersion of the signal in the delay domain. The RMS DS $\sigma_{\tau }$ can be denoted as

(28) $$\sigma_{\tau }=\sqrt{\sum_{k=1}^{K\left(t\right)}\sum_{l_{k}=1}^{L_{k}}P_{pq,l_{k}}\left(t\right){\left(\tau_{c}\left(t\right)+\tilde{\tau }\right)}^{2}-{\left(\sum_{k=1}^{K\left(t\right)}\sum_{l_{k}=1}^{L_{k}}P_{pq,l_{k}}\left(t\right)\left(\tau_{c}\left(t\right)+\tilde{\tau }\right)\right)}^{2}}$$

where $\tau_{c}$ can be denoted as

(29) $$\tau_{c}\left(t\right)=\frac{D_{p,l_{k}}^{T,MB}\left(t\right)+D_{q,l_{k}}^{R,MB}\left(t\right)}{c}.$$

## 4. Numerical Results and Analysis

For this section, some key statistical properties of the channel model, related to the Tx and Rx, were analyzed and compared. The moving trajectories of the Tx, the Rx, and the clusters are presented. Furthermore, the influences of different trajectories on the statistical properties of the channel model were further investigated.

The impact of motion trajectory on the PDP in the simulation was supposed to be observed, so the number of Tx and Rx antenna were set to one. The trajectory of the UAV was mainly determined by the speed $V_{P}$ and angle, where the angle was controlled by the azimuth angle α and by the elevation angle γ, and the random change of trajectory was realized by setting the random change of the two angles. The ST mobility model was used to achieve smooth turns in the RX trajectories, where $\delta_{s}^{2}$ were used to control the smoothness of the turns. A small variance $\sigma_{s}^{2}$ indicated a greater likelihood of a very large turn radius, which would show a straighter trajectory. Setting $\sigma_{s}\to 0$ meant the generation of a trajectory that was close to a straight line. The time interval $\tau_{i}$ represented the length of time that the Rx moved along the fixed steering center. Smaller time intervals meant that the trajectory would change turn centers frequently, which could lead to wavy trajectories. As shown in Figure 3a, a Markov chain was used to create the Tx trajectories, the α was random, and the γ was set as π/8 and π/3 for simulating the random flight. There are three trajectories shown in Figure 3b. For smooth-turning trajectories, the $\sigma_{s}^{2}$ was set to 0.007 m${}^{-1}$, and the time interval was set to 4 s.

**Figure 4:**
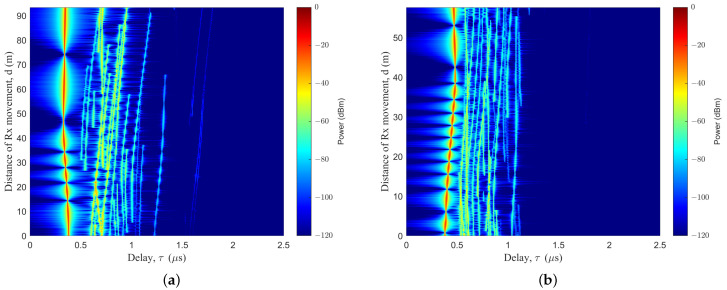
Time-variant PDP, based on different Tx moving trajectories for the MB case: (**a**) Tx for trajectory III, and Rx for trajectory I; (**b**) Tx for trajectory I, and Rx for trajectory I.

In the simulation of the MB case, the basic parameters were set as follows: $f_{c}$ = 3.5 GHz; $v_{UAV}$ = $v_{R}$ = 5 m/s, $a_{UAV}$ = $a_{R}$ = 0 m/s${}^{2}$. As shown in Figure 4a, the Tx trajectory was set as a line, to emulate the level-flight trajectory; a Tx trajectory based on a Markov chain was generated, as shown in Figure 4b. Based on the ST mobility model, the Rx trajectory was set as trajectory I, as shown in Figure 4a; the Rx trajectory was set as trajectory II, as shown in Figure 4b. From the time-variant PDP, there is a bright red line in Figure 4, representing the LoS path. Some subpaths were arranged to the right of the LoS path. The delay of the LoS path reflected the relative distance between the Tx and the Rx. From the PDP diagram, we can see the drift of power, the delay, and other parameters, as well as the birth and death of clusters. The changes of multiple sub-paths reflected the influence of the changes of the scattering environment on the signal during transmission. The random trajectory in dual-mobility had a greater degree of curvature and a greater time delay than the linear trajectory from the PDP diagram. The results indicated that the trajectories of the Tx would have effects on the PDP.

**Figure 5:**
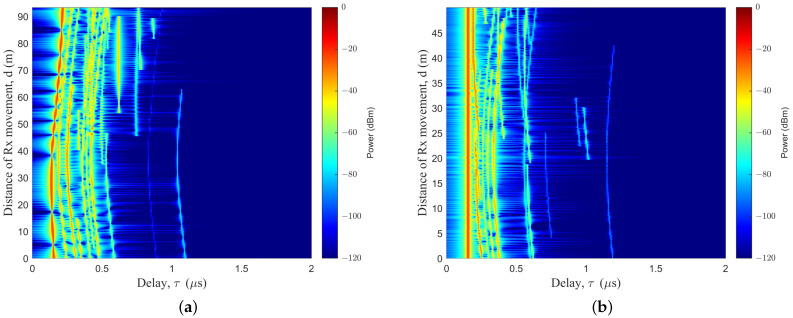
Time-variant PDP, based on different Rx moving trajectories for the SB case: (**a**) Tx for trajectory III, and Rx for trajectory II; (**b**) Tx for trajectory III, and Rx for trajectory III.

As shown in Figure 5, a simulation of a PDP diagram for the SB case was created. The trajectory of the cluster was assumed to be the same as that of the Rx during the simulation. In order to simulate urban transportation emergency communication scenarios, there were a large number of vehicles blocking the vehicles at the Rx, with the same vehicle trajectory.

**Figure 6:**
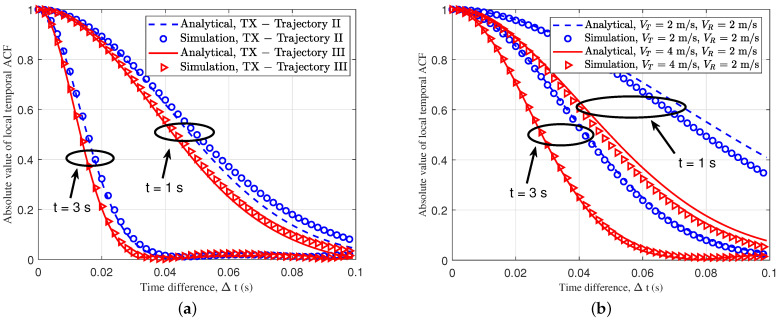
Temporal ACFs of different trajectories and velocities for the MB case ($f_{c}$ = 2.5 GHz): (**a**) Rx for trajectory III ($V_{T/R}$ = 4 m/s, $a_{T/R}$ = 3 m/s${}^{2}$); (**b**) Tx for trajectory I, and Rx for trajectory II ($a_{T/R}$ = 1 m/s${}^{2}$).

Figure 6 shows the temporal ACF. A temporal ACF can reflect the stationary characteristics of the AG channel. The time variation of the temporal ACF was caused by the time-variant MPCs between the Tx and the Rx, due to the motion of the UAV and the ground BS. For the simulated parameters of Figure 6a, the UAV speed was 4 m/s, the acceleration was 3 m/s${}^{2}$, and the Rx speed was 4 m/s. The random track of the Tx was formed by a Markov chain, and the Rx trajectory was set as linear. For the simulated parameters of Figure 6b, the UAV speed was 4 m/s or 2 m/s, the acceleration was 1 m/s${}^{2}$, and the Rx speed was 2 m/s, using the same trajectory. The Tx trajectory was random, based on a Markov chain, and the Rx trajectory was based on the ST mobility model. Through the simulation, we acquired the analysis results and the simulation results, which were well-fitted. With the flight of the UAV, and the movement of the ground Rx, the ACF curve at *t* = 3 s, under the two trajectories, became steeper, as shown in Figure 6a, which shows that the channel became more complex, compared to the ACF curve at *t* = 1 s. Due to the movement of the UAV and the vehicle, the arrival and departure angles of the MPCs changed with time *t*, which led to the non-stationarity of the channel. We found that the ACF curve of the linear trajectory was flatter, and that the channel was more stable than the random trajectory, which was due to the channel instability caused by the large angular variation of the random trajectory.

**Figure 7:**
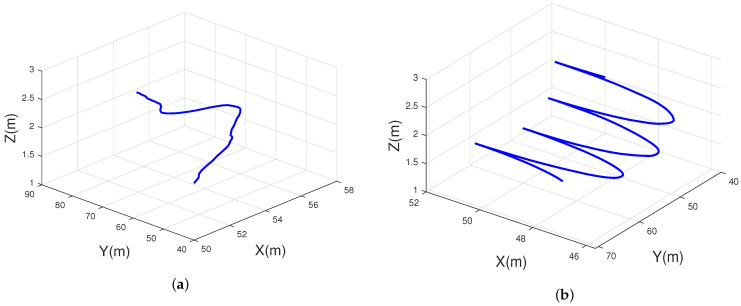

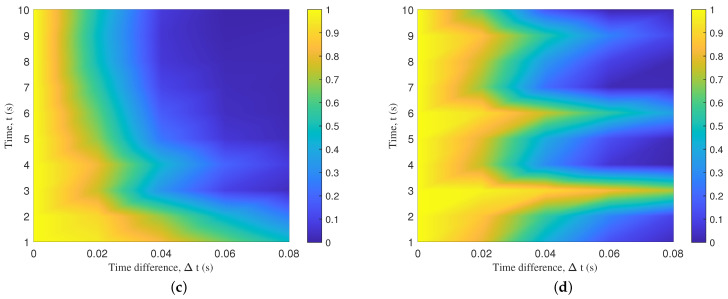
3D temporal ACFs of different trajectories (moving time for 10 s, $V_{T}$ = $V_{R}$ = 5 m/s, $a_{T}$ = $a_{R}$ = 0 m/s${}^{2}$): (**a**) Case I: Tx for trajectory II, based on a Markov chain, and Rx for trajectory III; (**b**) Case II: Tx for random trajectory I, based on a Markov chain, and Rx for trajectory III; (**c**) 3D ACF based on Case I; (**d**) 3D ACF based on Case II.

**Figure 8:**
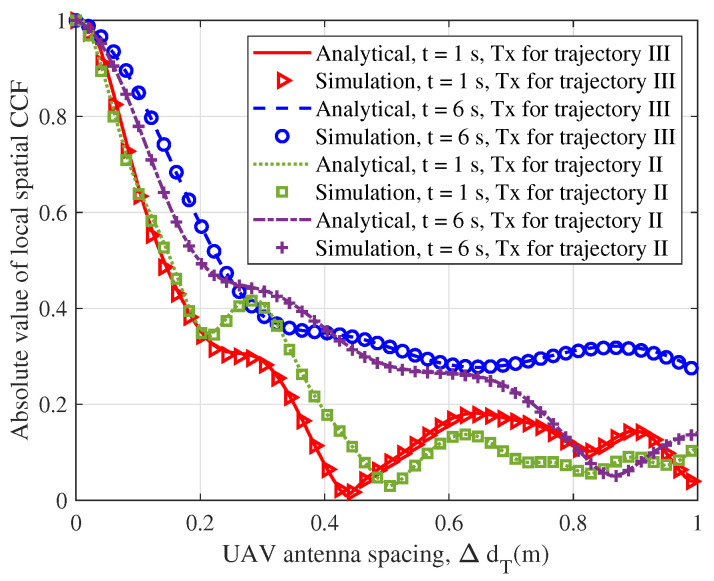
Comparison between the spatial CCFs of the analytical model and those of the simulation model, with different trajectories and different time instants ($V_{T}$ = 35 m/s, $\beta_{E}^{R}$ = 0).

**Figure 9:**
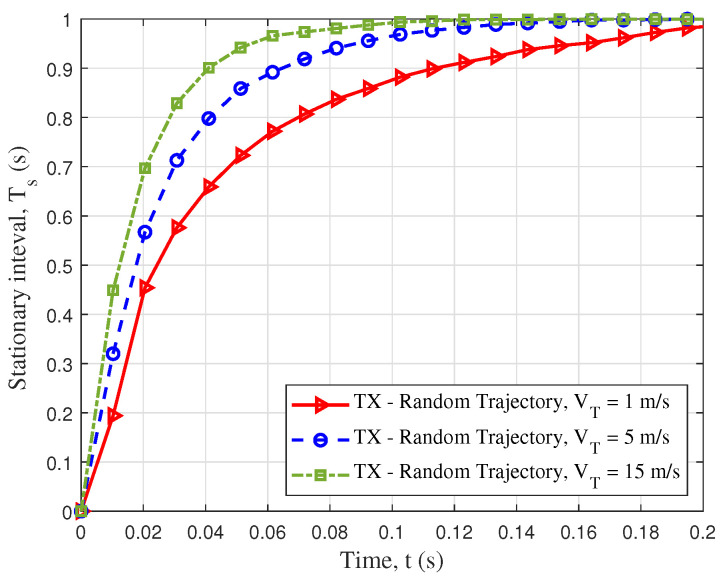
CDF of stationary interval, based on different velocities of the Tx and the Rx for trajectory III ($V_{R}$ = 2 m/s, $a_{T}$ = $a_{R}$ = 0 m/s${}^{2}$).

As for the simulated parameters of Figure 6b, we kept the Tx and the Rx trajectories unchanged, and the accelerations at both ends were set to 1 m/s${}^{2}$. Through simulation, it was found that when the $v_{UAV}$ was large, the ACF curve would be steeper; therefore, we think that higher velocity of the UAV would lead to more drastic changes in the trajectory, which would lead to a decline in the stationarity of the channel. Figure 7 shows the 3D temporal ACFs of different trajectories. It shows the temporal ACF at the moment of flight of the drone in 10 seconds. To better show the relationship between the trajectory and temporal correlation, Figure 7a,b show the 3D trajectories projected to the XoY plane. The change in angle caused a decrease in the channel stationarity, and the characteristics are well reflected in Figure 7c,d. Figure 8 shows the spatial CCF of different distances; the CCF could reflect the spatial correlation of the antenna array. For the simulation parameters, the Rx trajectory was set as an unchanged static point, and the Tx trajectories were set as trajectory II and trajectory III, which simulated random slanting flight and linear level flight. Different trajectories of a UAV can lead to different spatial correlations. From the figure, two kinds of trajectories led to different spatial correlations in different time instants. Compared to the level flight, the height of the random slanting flight was larger; therefore, the slanting flight had the lower spatial correlation. Figure 9 shows the CDF of stationary intervals based on multiple velocities. In the Figure, at different velocities, the stationary intervals of random trajectories decreased with increasing speed.

**Figure 10:**
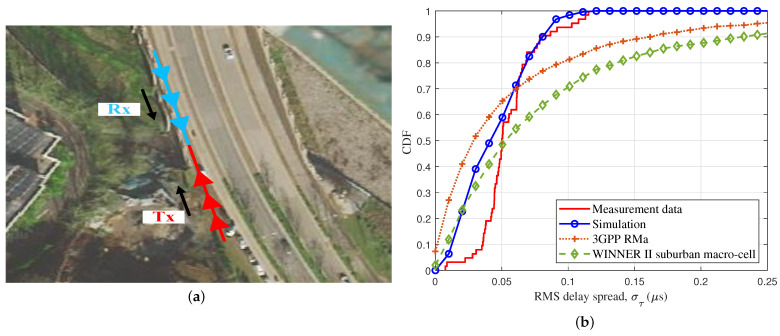
The CDF of the RMS DS with simulation and measurement data ($v_{UAV}$ = 5 m/s, $v_{vehicle}$ = 5 m/s, $f_{c}$ = 3.6 GHz): (**a**) satellite cloud image of the measurement site; (**b**) CDF of the RMS DS, based on measurement.

The channel measurements were conducted, taking into consideration the multi-mobility multi-trajectories of the Tx and the Rx. The satellite views and related trajectories are shown in Figure 10a. The measurement scene of the actual data was located on a bridge by Xingzhong Road. At the time of measurement, there were a large number of trees on both sides of the road, and a large number of vehicles moving. The Rx and the Tx were 50 m apart, both at a speed of 5 m/s, moving in opposite directions. The motion trajectories are shown as blue and red lines in Figure 10a. The measurement equipment consisted of an airborne part and a ground part. The airborne part equipment consisted of a universal software-defined radio peripheral (USRP), an omni-directional antenna, a microcomputer, and a small power supply. The ground part equipment consisted of a USRP, an omni-directional antenna, a computer, and an outdoor power supply. Two USRPs and two omni-directional antennas were used for signal transmission and reception. A high-order pseudo-noise (PN) sequence was transmitted from the Tx end to the Rx end. Figure 10b shows the CDF of the RMS DS, with measurement data, simulation data, and reference model. The measurement result showed that our proposed model can mimic real multi-mobility multi-trajectory channel characteristics. Compared to the WINNER II suburban macro-cell [36] and the 3rd Generation Partnership Project (3GPP) [37] RMa, our model fits better to the actual measured data in the field.

## 5. Conclusions

In this article, a novel multi-mobility UAV channel model, based on different 3D dynamic trajectories, is proposed. For this study, a Markov chain was introduced, to realize the randomness of the Tx, and an ST mobility model was used for generation of the Rx trajectory, to simulate the actual situation of UAV-assisted emergency communications. Based on the multi-mobility multi-trajectory model, the typical statistical properties of the proposed channel model—such as time-variant PDP, temporal ACF, spatial CCF, stationary interval, and RMS DS—were investigated. Moreover, the RMS DS of our proposed model was validated by the measurement data. The results proved that the trajectories of both UAVs and ground vehicles, and the trajectories of moving vehicles around the Rx, had an impact on the PDPs. For the same trajectory of the Tx, the time-variant ACFs presented different changing trends according to different Rx trajectories. For the same trajectories of the Tx and the Rx, the time-variant ACF had a faster decline with the increasing of UAV velocity. The variation of the Rx trajectory had little effect on the CCFs. Frequent movements of UAVs, as well as ground vehicles, can affect the stationarity of the channel in time as well as in space domains. Our future work will increase the scope of application of the trajectory at the ground Rx and UAV Tx ends, and will make the model more consistent with real scenarios.

[custom]

## Figures and Tables

**Table 1:** Definition of channel model parameters.

Symbol	Definition
K(t)	The number of clusters
$L_{k}$	Rays number of the *k*th cluster
$C_{k}^{A}\left(t\right)/C_{k}^{Z}\left(t\right)$	Coordinates of the center of clusters $C_{k}^{A}/C_{k}^{Z}$
$C_{l_{k}}^{A}\left(t\right)/C_{l_{k}}^{Z}\left(t\right)$	Coordinates of the scattering points of the *l*th ray in the *k*th cluster
$\phi_{A,l_{k}}^{T}\left(t\right)/\phi_{E,l_{k}}^{T}\left(t\right)$	AAoD and EAoD of the *l*th ray in the *k*th cluster
$\phi_{A,l_{k}}^{R}\left(t\right)/\phi_{E,l_{k}}^{R}\left(t\right)$	AAoA and EAoA of the *l*th ray in the *k*th cluster
$\phi_{A,LoS}^{T}\left(t\right)/\phi_{E,LoS}^{T}\left(t\right)$	AAoD and EAoD of the LoS ray
$\phi_{A,LoS}^{T}\left(t\right)/\phi_{E,LoS}^{T}\left(t\right)$	AAoA and EAoA of the LoS ray
$\theta_{l_{k}}^{T}$	Initial phases subject to uniform distribution in (0, 2π]
$\kappa_{l_{k}}$	Cross-polarization power ratio
$D_{l_{k}}^{T,MB}\left(t\right)/D_{l_{k}}^{R,MB}\left(t\right)$	Distance of the *l*th ray between Tx/Rx and clusters for the MB case
$D_{pq}^{LoS}\left(t\right)$	Distance of the LoS path between the *p*th antenna and the *q*th antenna
$A_{p}^{T}\left(t\right)/A_{q}^{R}\left(t\right)$	Coordinates of *p*th Tx or *q*th Rx antenna
$C_{l_{k^{\prime }}^{\prime }}\left(t\right)$	Coordinates of the single cluster for the SB case
$D_{p,l_{k^{\prime }}^{\prime }}^{T,SB}\left(t\right)$/$D_{q,l_{k^{\prime }}^{\prime }}^{R,SB}\left(t\right)$	Distance of the *l*th ray between the Tx/Rx and the single cluster for the SB case
$V_{P}\left(t\right)$	The speed vector of the Tx
$V_{Q}\left(t\right)$	The speed vector of the Rx

## Data Availability

Not applicable.
